# Unraveling incompatibility between wheat and the fungal pathogen *Zymoseptoria tritici* through apoplastic proteomics

**DOI:** 10.1186/s12864-015-1549-6

**Published:** 2015-05-08

**Authors:** Fen Yang, Wanshun Li, Mark Derbyshire, Martin R Larsen, Jason J Rudd, Giuseppe Palmisano

**Affiliations:** Department of Plant and Environmental Sciences, University of Copenhagen, 1871 Frederiksberg C, Denmark; BGI-tech, BGI, 518083 Shenzhen, China; Department of Plant Biology and Crop Science, Rothamsted Research, Harpenden Hertfordshire, AL5 2JQ United Kingdom; Department of Biochemistry and Molecular Biology, University of Southern Denmark, 5230 Odense M, Denmark; Present address: Institute of Biomedical Science, Department of Parasitology, University of São Paulo, 05508-900 São Paulo, Brazil

**Keywords:** Anti-oxidative stress, Apoplastic proteomics, Plant resistance, *Zymoseptoria tritici*

## Abstract

**Background:**

Hemibiotrophic fungal pathogen *Zymoseptoria tritici* causes severe foliar disease in wheat. However, current knowledge of molecular mechanisms involved in plant resistance to *Z. tritici* and *Z. tritici* virulence factors is far from being complete. The present work investigated the proteome of leaf apoplastic fluid with emphasis on both host wheat and *Z. tritici* during the compatible and incompatible interactions.

**Results:**

The proteomics analysis revealed rapid host responses to the biotrophic growth, including enhanced carbohydrate metabolism, apoplastic defenses and stress, and cell wall reinforcement, might contribute to resistance. Compatibility between the host and the pathogen was associated with inactivated plant apoplastic responses as well as fungal defenses to oxidative stress and perturbation of plant cell wall during the initial biotrophic stage, followed by the strong induction of plant defenses during the necrotrophic stage. To study the role of anti-oxidative stress in *Z. tritici* pathogenicity in depth, a YAP1 transcription factor regulating antioxidant expression was deleted and showed the contribution to anti-oxidative stress in *Z. tritici*, but was not required for pathogenicity. This result suggests the functional redundancy of antioxidants in the fungus.

**Conclusions:**

The data demonstrate that incompatibility is probably resulted from the proteome-level activation of host apoplastic defenses as well as fungal incapability to adapt to stress and interfere with host cell at the biotrophic stage of the interaction.

**Electronic supplementary material:**

The online version of this article (doi:10.1186/s12864-015-1549-6) contains supplementary material, which is available to authorized users.

## Background

The ascomycete fungus *Zymoseptoria tritici* causes Septoria tritici blotch (STB) in wheat, a foliar disease that poses a significant threat to global food production. Leaf penetration occurs by means of fungal hyphae emerging from geminating, surface-attached spores that enter via stomata. The fungus has a slow intercellular biotrophic symptomless growth, for typically up to 10 days, as hyphae extend in close contact with mesophyll cells, probably utilizing lipid and fatty acid stores for growth [1,2]. Subsequently, the fungus suddenly switches to the necrotrophic growth associated with leakage of nutrients from dying plant cells into the apoplastic spaces, an increase in fungal biomass, enhanced signaling, metabolism and defense responses in host, the appearance of lesions on the leaf surface, and the collapse of the plant tissue [3,4]. Disease transition and appearance of symptoms have been suggested to be triggered by fungal small protein effectors secreted into apoplast [2-4]. Differing from many other phytopathogenic fungi, *Z. tritici* does not form any specialized penetration or feeding structures and remains strictly apoplastic throughout the entire infection cycle.

The plant apoplast is potentially important as a bridge that perceives and transduces signals from the environment to the symplast. Under stress conditions, complex mechanisms, including accumulation of reactive oxygen species (ROS) and changes in the synthesis of extracellular proteins, are activated in the apoplast as a first line of defenses. The secreted plant apoplastic proteins predominantly represent functional categories associated with carbohydrate metabolism, cell wall metabolism, defense, and programmed cell death [5]. As an apoplast-inhabiting fungus, *Z. tritici* need to acquire apoplastic nutrients, shape the plant cell structures, and overcome the activated apoplastic defenses to survive, possibly via secretion of effectors involved in detoxification of defense-related molecules as well as protection against recognition by the plant. This highly dynamic compartment serves as the molecular battlefield that contributes to the success of infection or plant resistance.

Given the crucial role of leaf apoplast in wheat-*Z. tritici* interaction, it is of particular interest to investigate the molecular basis underlying plant apoplastic immunity and the counter defenses that *Z. tritici* evolves at different growth stages. Although there have been advances in understanding mechanisms of wheat responses to *Z. tritici*, involving programming cell death, ROS accumulation, activation of signal transduction, transport and energy metabolism, expression of a broad spectrum of pathogenesis-related (PR) proteins, antioxidants and jasmonic acid biosynthesis genes, and production of small signaling and defense compounds [1,2,4,6-10], well-characterized systematic apoplastic responses to *Z. tritici* in wheat, which are essential for determining the plant fate, are currently lacking.

Considerable studies have been performed to understand *Z. tritici* gene functions, mainly focusing on the necrotrophic growth due to low fungal biomass hardly detectable at the biotrophic stage. Until recently, the emerging high throughput ‘omics’ and sequencing technologies partly address the issue and enable the discovery of several fungal genes and proteins expressed at different growth stages [2,4,10]. Compared to uncovering the expression of *Z. tritici* genes including the genes encoding cell-wall-degrading enzymes (CWDEs), ROS-scavenging enzymes and putative effector proteins such as LysM as well as the production of the secondary metabolites during the compatible interaction [1-4,11], *Z. tritici in planta* proteins, particularly secreted protein effectors, have not been fully explored at a systematic level. The only proteomic report identified thirty-one proteins and five phosphoproteins of *Z. tritici* mainly involved in basic cellular machinery and signaling at the biotrophic stage of the compatible and incompatible interactions [10]. This study revealed a similarity in fungal protein profiles between two interactions, possibly due to the fact that analysis of whole inoculated leaves resulted in the dominance of most abundant plant and fungal proteins, which largely diluted the information about low abundant fungal proteins likely essential for pathogenicity. A deeper insight into *Z. tritici*-specific strategies of colonization can be achieved by a detailed analysis of host apoplast for the enrichment of fungal identifications, in particular secreted molecules.

The expression of genes encoding ROS-scavenging enzymes is one of the multiple defense systems evolved by *Z. tritici* to thrive in the oxidative apoplastic environment. The YAP1 transcription factor is one of the most important determinants of oxidative stress responses, responsible for transcriptional activation of oxidative stress-associated genes in fungi [12]. YAP1 undergoes a conformational change due to the formation of disulfide bonds upon exposure to oxidative stress and is thus transported from the cytoplasm into the nucleaus to activate gene transcription. YAP1-mediated detoxification of ROS is essential in the virulence of many pathogenic fungi including biotroph *Ustilago maydis*, necrotroph *Alternaria alternate* and human pathogen *Candida albcans* [13]. However, the role of YAP1 in oxidative stress responses during *Z. tritici* colonization is poorly defined.

In order to gain further insights into host resistance and *Z. tritici* pathogenicity, we explored the proteome from apoplastic washing fluid (AWF) with emphasis on the host and the pathogen during the compatible and incompatible interactions. This is a perspective focusing on the direct battle ground and differing from the previous genome-wide studies on plant-*Z. tritici* interaction. The analysis uncovers apoplastic regulatory networks that shape the aspects of the plant physiology in response to the fungus and the principles of fungal modulation of apoplastic immunity. The combat between the host and *Z. tritici* through the activation of their defense mechanisms at the biotrophic stage likely determines their fates. Our data have also expanded on the current models of fungal apoplastic proteome.

## Results

### Wheat proteins from AWF in response to *Z. tritici*

It was difficult to avoid the leakage of symplastic compounds in leaf apoplast during the extraction, particularly from the dying leaf cells in the necrotrophic phase. In order to obtain the important apoplastic components at the necrotrophic stage but avoid severe symplastic contamination, we sampled the leaves at the early time point of the necrotrophic growth (14 days after inoculation, 14 dai) when immature asexual sporulation structure started to form. Malate dehydrogenase (MDH) was used as a cytosolic enzyme marker to evaluate the level of contamination. MDH activities in AWF contained max. 1% of those from the whole leaf extracts in all samples (Additional file 1). The ratio below 1% is considered as little damage to the cells and is acceptable for plant apoplast studies [14]. Approx. 1% MDH activity related to the total leaf extracts was also observed in the apoplastic tissue of ryegrass leaves during senescence [15], and of *V. longisporum*-infected oilseed rape leaves [16] and *A. thaliana* [17]. This recurrent and unavoidable cytoplasmic contamination has already been highlighted by several previous studies of plant apoplast [18-22]. Therefore, we considered apoplastic proteins were enriched in the AWF in the present study.

We applied a mass spectrometry (MS)-based shotgun quantitative proteomics using isobaric tags for relative and absolute quantitation (iTRAQ) labeling. This approach has been developed to analyze the proteome and phosphoproteome of *Z. tritici*-inoculated wheat leaves [10]. Missing reporter ions of the peptide from some of the samples in the MS/MS spectrum can occur in the iTRAQ-based proteomics due to the incomplete labeling and inefficient fragmentation of the tags, which results in no quantitative ratio. Therefore, the criterion defined for the reliable quantified proteins was that proteins had to be identified in at least two biological replicates with quantitative ratio. This resulted in the identification of 2122 and 2071 wheat proteins and quantification of 607 and 575 proteins in Stakado (resistant cultivar) and Sevin (susceptible cultivar), respectively (Additional file 2). Not surprisingly, cytosolic proteins such as Rubisco and ribosomal proteins were identified in all the samples by this highly sensitive MS approach, which were eliminated for the further analysis.

Based on the selection criteria, 45 proteins in Stakado and 100 proteins in Sevin were found to change in abundance in response to the fungus (Additional file 3). Eleven were detected in both cultivars. Of the regulated proteins, 42% and 60% from Stakado and Sevin were predicted with signal peptides using SignalP program, respectively. Different percentages of proteins with predicted signal sequences were found in the secretome of soybean (65%), grapevine (66%), *Arabidopsis* (47%) and rice (37%) [18]. There were 43 and 96 differentially expressed proteins identified from Stakado and Sevin whose corresponding transcripts were identified from the previous transcriptome dataset, respectively [4].

**Figure 1:**
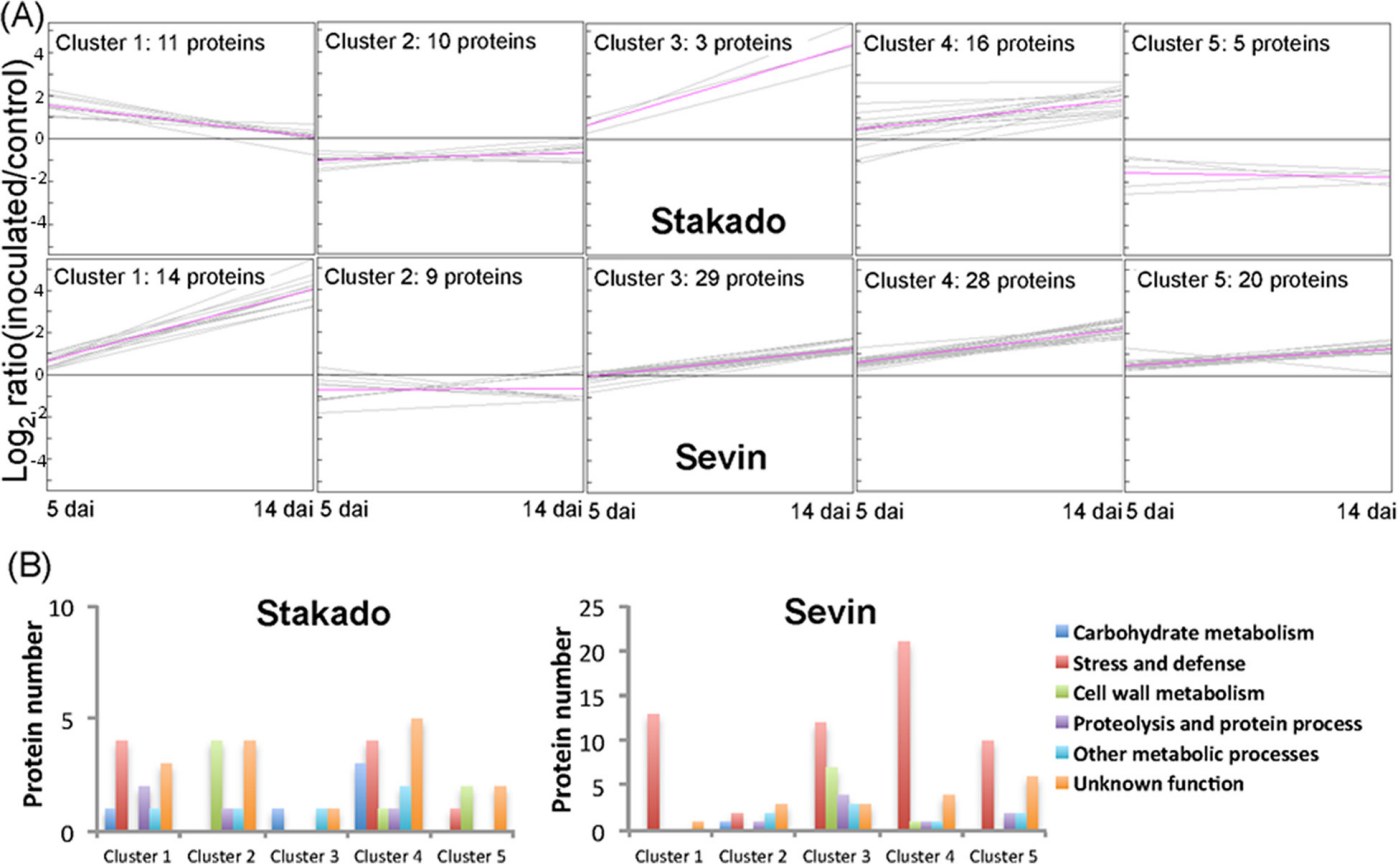
Differentially expressed plant proteins identified from AWF. AWF was isolated from the leaves of wheat cvs. Stakado (resistant) and Sevin (susceptible) inoculated with *Z. tritici* or water (control) at 5 and 14 dai. Three biological samples were prepared. The proteins from AWF were labeled with iTRAQ and subjected to LC-MS/MS analysis. Differentially expressed plant proteins were analyzed by expression clustering **(A)** and functional classification **(B)**.

Expression cluster analysis revealed that a substantial number of proteins were up-regulated only at the necrotrophic stage (14 dai) in contrast to minor changes at the biotrophic stage (5 dai) in Sevin, whereas proteins could be up- or down- regulated at both time points in Stakado (Figure 1A). The major proteins in each cluster were annotated in carbohydrate metabolism, stress, defense, cell wall metabolism, protein process, and other metabolic processes (Figure 1B). In Stakado, at either time point, the fungus caused a significantly increased expression of several carbohydrate metabolic proteins involved in glycolysis (*i.e.*, 2,3-bisphosphoglycerate-independent phosphoglycerate mutase and phosphoglycerate kinase), pentose phosphate pathway (*i.e.*, 6-phosphogluconate dehydrogenase) and TCA (*i.e.*, aconitate hydratase), as well as stress/defense-related proteins including PR-1, PR-2, PR-3, peroxidase 3, heat shock protein and glycolate oxidase (clusters 1, 3 and 4), in contrast to a decreased expression of cell-wall metabolic proteins including α-L-arabinofuranosidase, β-D-glucan exohydrolase, 1,3(4)-β-glucanase, and β-D-xylosidase (cluster 2 and 5). The homologs of aconitate hydratase, 2,3-bisphosphoglycerate-independent phosphoglycerate mutase and phosphoglycerate kinase were also identified in the study of poplar apoplastic proteome [5]. Additionally, a 1-deoxy-D-xylulose 5-phosphate reductoisomerase responsible for terpenoid biosynthesis displayed a strong accumulation (11-fold change) at 14 dai, indicating the possible involvement of terpenoid in wheat resistance to *Z. tritici*. The pathogen-induced expression of terpenoid synthase gene and production of diterpenoid phytoalexins have been seen in maize and rice [23,24]. Moreover, integration of proteome and previous transcriptome datasets revealed that the majority of regulated proteins during the incompatible interaction did not change at transcription level in the compatible interaction. This result further emphasizes an important role of these proteins in plant resistance to *Z. tritici*. In Sevin, up-regulated proteins at 14 dai consisted of proteins involved in protein process, cell wall metabolism, stress, and defense, including a cold acclimation induced protein, peroxidases and numerous PR-proteins belonging to PR families 1, 2, 3, 4, 5, and 17 (clusters 1, 3, 4 and 5). A good correlation between protein and transcript regulation at the necrotrophic stage was observed, indicating the great amplitude of activation of host defense responses. On the other hand, most proteins that did not change in abundance at the biotrophic stage of the compatible interaction were transcriptionally suppressed, suggesting a complexity of molecular mechanisms in host plant triggered by *Z. tritici* biotrophic growth.

**Figure 2:**
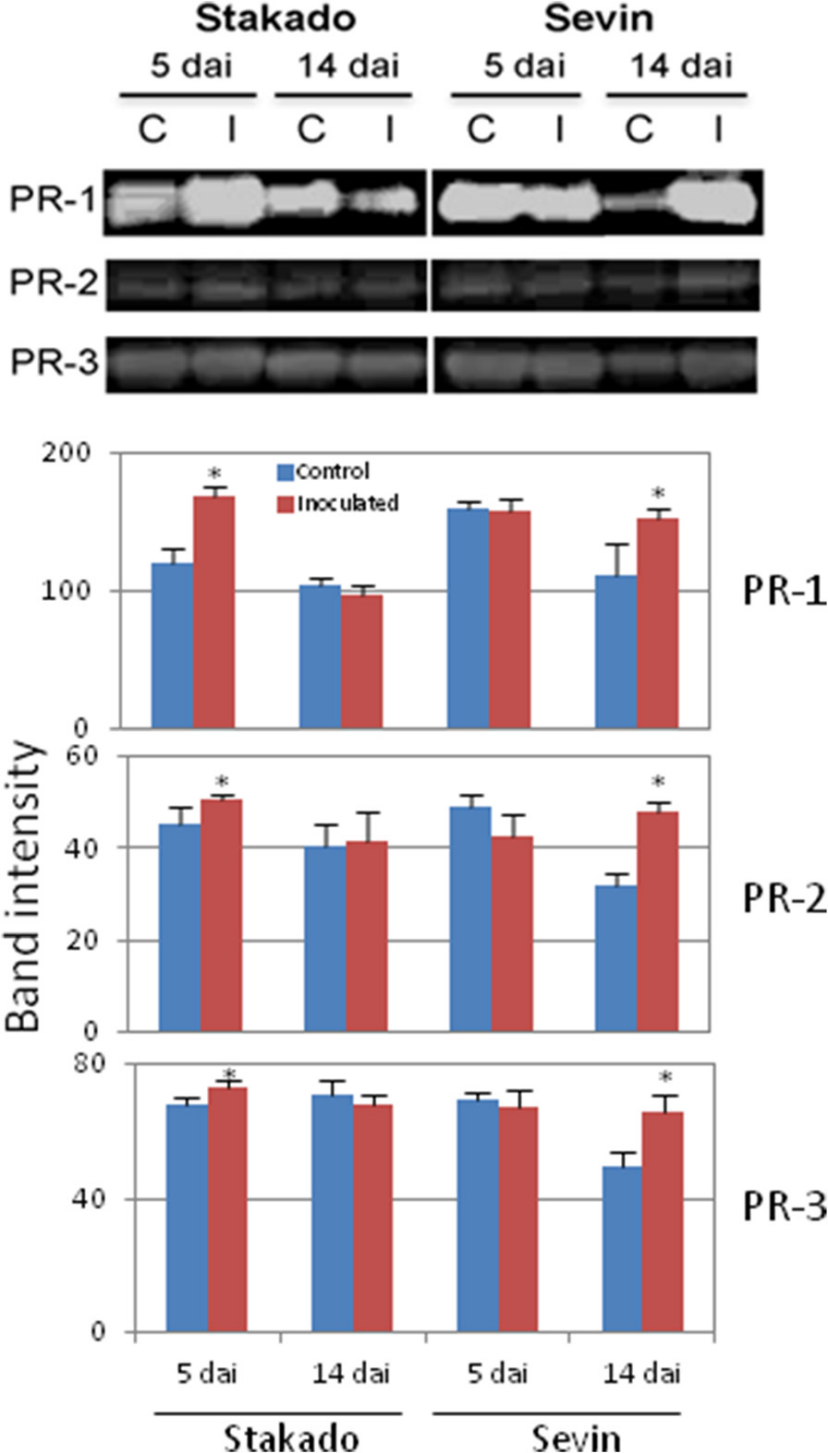
Western blotting validation of proteome data. Protein was extracted from three biological replicates of control (C) and *Z. tritici*-inoculated (I) wheat cvs. Stakado (resistant) and Sevin (susceptible) at 5 and 14 dai and used in western blot analysis. The representative membranes of PR-1, PR-2 and PR-3 protein expression are shown. Quantification of signals on the membranes was performed by using ImageJ program based on three biological samples. The asterisks indicate significant differences in signal intensity (*P* ˂ 0.05) between control and inoculated samples.

In order to validate our proteomics data, western blot analysis of PR-1, PR-2, and PR-3 proteins was conducted with three biological replicates of AWF samples. In agreement with the proteomics analysis, the abundance of PR-1, −2, and −3 increased slightly at the early biotrophic stage of the incompatible interaction and markedly at the late stage of the compatible interaction (Figure 2).

### Fungal proteins identified from inoculated leaf AWF

It is difficult to distinguish whether the protein is produced by the plant, the fungus or both in a plant-fungus interaction system, when the peptide matches to the protein sequences from both host and pathogen databases. Therefore, we have showed the proteins with peptides confidently identified from fungal database only, since the proteins identified from both databases are more likely of plant origin due to high biomass ratio of plant to the fungus. Twenty-four and thirty-one fungal proteins were identified from the inoculated Stakado and Sevin, respectively (Table 1). There were eighteen proteins whose corresponding transcripts were identified from the previous RNA-seq-based transcriptome dataset (Table 1, Additional file 4). Twelve proteins had an N-terminal signal peptide sequence predicted by SignalP, indicating they could be secreted proteins. The identified fungal proteins with known functions could be assigned to biological processes, encompassing metabolism, degradation of host cell wall, signaling, stress, defense, transport, cell mobility, cell component organization, and cell wall degradation and remodeling. Despite similar functional categories of fungal proteins and nine proteins identified from both inoculated cultivars, the overviews of fungal protein profiles were distinct between the compatible and incompatible interactions (Table 1). Apart from the categories of primary carbohydrate, amino acid and protein metabolisms regarded as no significant changes between two interactions, the fungus seemed to preferentially express the proteins involved in signal transduction, transport, and cell wall remodeling during the incompatible interaction, whereas the proteins involved in nucleic acid metabolism, transcription, perturbation of plant cell wall, anti-oxidative stress, and cellular component organization could only be identified in the compatible interaction.

**Table 1:** ***Z. tritici*** **proteins identified from** ***Z. tritici*** **-inoculated wheat leaves**

		**Ratio (14 d/ 5 d)**			
**ID**	**Annotation**	**S**	**SK**	**SV**	**Biological process**
91239	Myosin			1.36	Cell mobility
**105948**	Actin		1.15	1.11	Cell mobility
110409	Myosin	Y	1.17	1.33	Cell mobility
45905	Chitinase		0.52		Cell wall remodeling
86354	SWI/SNF chromatin remodeling complex protein			N/A	Cellular component organization
87313	Rrp15p domain-containing protein			1.04	Cellular component organization
68922	Glycoside hydrolase family 62	Y		4.18	Degradation of plant cell wall
**70396**	α-L-arabinofuranosidase B	Y		5.67	Degradation of plant cell wall
99970	β-glucosidase	Y		2.70	Degradation of plant cell wall
44922	N6 adenine-specific DNA methylase			N/A	Metabolism
46697	ATPase		0.81		Metabolism
52059	Formate-tetrahydrofolate ligase		N/A	1.05	Metabolism
**55968**	Triosephosphate isomerase		1.63	1.38	Metabolism
64923	Glucose/ribitol dehydrogenase			7.74	Metabolism
68338	Isopenicillin N synthase	Y	1.23		Metabolism
69333	Glycine dehydrogenase		0.67		Metabolism
73114	Hydantoinase/oxoprolinase			2.31	Metabolism
**74730**	ATP-citrate lyase/succinyl-CoA ligase		1.81		Metabolism
**76530**	D-isomer specific 2-hydroxyacid dehydrogenase	Y		N/A	Metabolism
**77172**	Aldo-keto reductase		1.37	1.70	Metabolism
96092	Trypsin	Y	1.80	0.78	Metabolism
**102177**	20S proteasome		1.58	0.99	Metabolism
**106055**	Aconitate hydratase		1.05		Metabolism
107787	Mediator16 domain-containing protein			1.86	Metabolism
**110230**	α -isopropylmalate/homocitrate synthase			1.35	Metabolism
65824	Serine/threonine-specific protein phosphatase			1.07	Signaling
**98343**	Ras GTPase		1.64	1.22	Signaling
99493	Ras GTPase		2.33		Signaling
**99564**	Calmodulin		1.02		Signaling
**110139**	14-3-3 protein		N/A		Signaling
**67250**	Catalase/peroxidase	Y		36.9	Stress and defense
**75170**	Chaperonin 60		0.81		Stress and defense
**99959**	Heat shock protein 90	Y		1.04	Stress and defense
**103593**	Copper/Zinc superoxide dismutase			4.33	Stress and defense
**105409**	Catalase/peroxidase			9.33	Stress and defense
**105895**	Heat shock protein 70			N/A	Stress and defense
77089	Adaptin			1.05	Transport
83550	Golgi transport complex		1.07		Transport
94552	Ca^2+^-modulated nonselective polycystin	Y	1.30		Transport
102764	Ankyrin		0.63		Transport
67764	Protein of unknown function DUF185			0.42	--
69789	Hypothetical protein	Y	0.64		--
90006	Hypothetical protein		0.94	1.33	--
92804	Hypothetical protein			0.75	--
103686	Hypothetical protein		0.45		--
109652	Membrane protein containing DUF221	Y		0.45	--

Fungal proteins were identified from wheat leaves of resistant cultivar Stakado (SK) and susceptible cultivar Sevin (SV) inoculated with *Z. tritici* at 5 and 14 days by LC-MS/MS. ID is protein accession number from Joint Genome Institute gene index for *Zymoseptoria tritici*. Proteins whose corresponding transcripts were identified from RNA-seq-based transcriptome dataset [4] are indicated in bold. The expression of these transcripts marked in bold is shown in Additional file 4. Y indicates protein identification containing a signal peptide examined by SignalP (S) (http://www.cbs.dtu.dk/services/SignalP/). The present ratios of protein abundance between 14 days and 5 days were calculated from at least two biological replicates by iTRAQ-117/iTRAQ-115 in labeling-based quantitative proteomics analysis. The ratios ≥ 2 or ≤ 0.5 were defined as significant change. N/A indicates the identified protein without quantitative data. No ratio value shown indicates the protein was not identified in the cultivar.

Furthermore, the changes in fungal protein expression at two time points were measured. A signaling-related protein Ras GTPase was found to increase the expression during the incompatible interaction. The predominant fungal proteins identified from the inoculated Stakado, did not significantly change in abundance, indicating the restraint fungal growth during the incompatible interaction. A hydantoinase, a glucose/ribitol dehydrogenase, three CWDEs and three ROS-scavenging enzymes strongly accumulated during the compatible interaction, suggesting an increase in carbohydrate metabolism, interfering with host cell and oxidative stress. By comparing the changes in fungal proteins and the corresponding transcripts from the previous study during the compatible interaction, it clearly illustrates a strong multilevel induction of the mechanisms of anti-oxidative stress and host cell wall perturbation in *Z. tritici*, the greater depth and sensitivity of detection of molecules by RNA-seq approach, and the tendency for proteomics methodologies to preferentially detect proteins of higher abundance (Additional file 4).

### *ZtYAP1* contributes to resistance to oxidative stress but is not required for fungal pathogenicity

**Figure 3:**
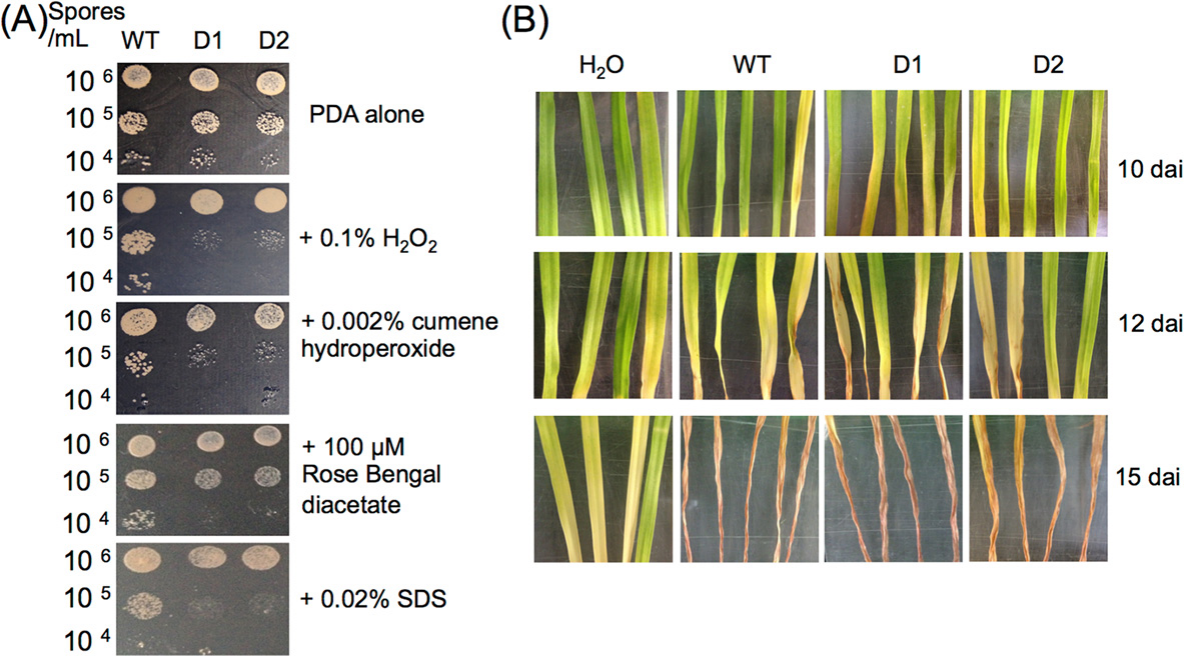
Characterization of *ΔZtYAP1* mutant. **(A)** *ΔZtYAP1* mutant is hypersensitive to oxidants *in vitro*. Sensitivity of *Z. tritici* wild type (WT) and *ΔZtYAP1* deletion strains D1 and D2 was determined by radial growth on PDA supplemented with oxidants or compounds as indicated. Results from one representative of two technical replicates are shown. **(B)** *ΔZtYAP1* mutant is as virulent as WT *in planta*. Fungal pathogenicity was assayed on wheat leaves spray-inoculated with spore suspension (1 x 10^6^ spores/mL) prepared from WT, D1, and D2 strains. Photos were taken at 10, 12 and 15 dai.

Since the fungus distinctively secreted antioxidants during the compatible interaction, a gene encoding transcription factor YAP1 activating the expression of genes encoding antioxidants was targeted for deletion to examine the role of anti-oxidative stress mechanisms in fungal pathogenicity. The *ZtYAP1* mutant slightly reduced growth on regular Potato Dextrose Agar (PDA) compared to wild type (Figure 3A). Growth of the mutant was strongly inhibited in the presence of H_2_O_2_, oxidizing compound cumene hydroperoxide, Rose Bengal diacetate generating singlet oxygen or SDS (Figure 3A). Additionally, the mutant retained normal formation and germination of conidia at a rate and magnitude similar to the wild type *in vitro* (data not shown).

To determine whether *ZtYAP1* gene is required for fungal pathogenicity and lesion development, conidial suspensions prepared from the wild type and the mutant were spray-inoculated onto detached susceptible wheat leaves. Disease symptoms on the leaves inoculated with the wild type or the mutant appeared at 10 dai and developed rapidly afterwards (Figure 3B). No clear difference in leaf lesion caused by the wild type and the mutant was observed throughout the entire infection process. The spore production of the mutant in 21-d-infected wheat leaves was approximately three times as high as that of the wild type.

## Discussion

Because of the economic importance as a major fungal pathogen of wheat, *Z. tritici* has emerged as model for studying plant-pathogen interaction. Understanding the molecular mechanisms underlying plant defenses and fungal pathogenicity is a prerequisite for understanding the host-pathogen interaction and can contribute to the development of new strategies of crop protection against pathogen. Thus, we have presented a detailed analysis of proteome from the battle ground apoplast between host wheat and *Z. tritici* during the compatible and incompatible interactions. A comparison of the apoplastic protein profiles of both host and the pathogen between two interactions allows us to gain further insights into such mechanisms.

The analysis of cellular location of protein identifications in the present study shows that approx. half of the differentially expressed plant proteins and fungal proteins from the AWF do not contain signal peptides. This could a consequence of cell lysis leading to the release of cytosolic proteins in the apoplast. Alternatively, evidences are emerging to support that proteins identified in the AWF without symplastic contamination and lacking signal peptides could be secreted by a non-classical secretory mechanism as described in bacteria and fungi [5,18,25]. They are named leaderless secreted proteins (LSPs). Some predicted intracellular proteins such as carbohydrate metabolic proteins found in the AWF can be in fact actively translocated into the extracellular space. Inventories of plant secretome reveal that LSPs may account for up to 50% of the whole leaf proteins identified in the extracellular fluid [25]. The secretome of plants submitted to stresses usually contains more LSPs than unstressed plants, which has been shown in the pathogen-infected maize [26] and salicylic acid-treated *Arabidopsis* cells [27]. With respect to the fungal secretome, investigation of culture supernatant of the fungus *Fusarium graminearum* has indicated that 30% of the protein identifications may be secreted but do not contain signal peptides [28].

A notable difference in the profiles of differentially expressed plant proteins identified from the AWF between compatible and incompatible interactions is the occurrence of regulation of the proteins implicated in carbohydrate metabolism, defense, and stress. It was very likely that the carbohydrate metabolic proteins identified in the AWF were translocated from intracellular space into the apoplast [25]. Plant defense against pathogens is known to be costly in terms of energy, assimilates, reducing equivalents, and carbon skeleton components that are provided by the primary metabolism [29]. Rapid mobilization and metabolism of the carbohydrates are important factors determining the outcome of plant-pathogen interactions. In the incompatible interaction between wheat and *Z. tritici*, it has been shown that accelerated sugar production associated with signal transduction cascades and expression of defense responses, occurs rapidly and intensively, compared to the compatible interaction [10]. Consistent with this, enhanced carbohydrate metabolism occurring in the intracellular space was accompanied by the activation of defense and anti-oxidative stress responses in the apoplast during the biotrophic stage of the incompatible interaction observed in the present study. By contrast, in the compatible interaction, apoplastic defenses were impeded or not activated at protein level and suppressed at transcription level during the establishment of biotrophic growth, but strongly triggered at both levels in the necrotrophic phase [2,4]. These results collectively suggest a contribution of rapid apoplastic defenses and energy metabolism to host resistance to *Z. tritici*.

Host plant cell wall metabolism and remodeling were also distinct in response to the fungus between two interactions. The plant cell wall represents a first line of defense against microbial pathogens since it is a preformed, passive physical barrier limiting access of pathogens and is actively remodeled and reinforced specifically at discrete sites of interaction with the pathogens [30]. During the incompatible interaction, the abundance of the plant proteins that hydrolyze the carbohydrate moieties of arabinogalactan, arabinoxylan, and other polysaccharides diminished, possibly preventing the degradation of cell wall polysaccharides, and thus stabilizing the plant cell wall. Similar results were seen in a study of rice root apoplastic proteome in response to salt stress [31]. Conversely, these proteins largely increased the abundance at the necrotrophic stage of the compatible interaction. On one hand, this phenomenon can result in the release of plant oligosaccharides triggering a set of basal defenses. On the other hand, in agreement with the previous transcriptome analysis [4], the necrotrophic growth of *Z. tritici* induces the high metabolic activity of host cell wall, giving rise to the loss of control of host cell permeability and host necrosis, which allows the fungus thrives on the dead plant.

Moreover, a large group of responsive proteins manifesting increased abundance in both interactions were peroxidases. In addition to catalyzing formation and the consumption of ROS, peroxidases have been implicated in the modification of plant cell wall structures [17]. Via oxidative cross-linking of monolignols, ploysacchraides, and cell wall proteins, peroxidases can reinforce the cell wall to restrict pathogen invasion. Accumulation of certain classes of peroxidases is involved in plant cell wall lignification that occurs as stress response to prevent the spread of fungal pathogens including *Verticillium* and *Melampsora* species [5,17]. Despite the up-regulation of diverse peroxidases as well as other defense-related protein like PR proteins in the necrotrophic phase of the compatible interaction, the velocity and magnitude of activated apoplastic defenses were apparently insufficient to prevent the necrotrophic growth of *Z. tritici*.

An important aspect in studying AWF of the plant challenged with the apoplast-inhabiting pathogens is to obtain the knowledge concerning the molecular compounds produced or secreted by the pathogens, which can provide the candidates essential for the pathogenicity. Here, proteomics analysis showed that approx. half of the fungal proteins whose corresponding transcripts were not identified in the previous RNA-seq-based transcriptome dataset [4]. Similar results were observed in the global analysis of transcript and protein levels across the *Plasmodium falciparum* lifecycle [32]. The little correlation between mRNA and protein abundance has been well demonstrated due to the regulation of transcription and translation, mRNA processing, mRNA stability and degradation, and protein modifications and turnover [4]. Furthermore, the bias in methodologies of RNA-seq and proteomics can result in the discordance between detected transcripts and proteins. Combined with the poor correlation between the regulation of host apoplastic proteins and corresponding transcripts at the biotrophic stage of the compatible interaction, the results strongly suggest that the integrated ‘omics’ studies are required to comprehensively understand the molecular processes during *Z. tritici* colonization of host wheat.

Based on the fungal protein identifications, there is little evidence for fungal nutrient acquisition from the plant throughout symptomless biotrophic colonization by *Z. tritici* in the incompatible and compatible interactions, which further supports the previous findings from transcriptome and proteome datasets [2,10]. Different protein profiles of *Z. tritici* between two interactions were observed, suggesting specific strategies of *Z. tritici* for a successful colonization. Most notably, signaling, transport and cell wall remodeling were particularly identified in the incompatible interaction, whereas genes and proteins involved in anti-oxidative stress and modification of plant cell wall were identified and induced during the compatible interaction [4]. Fungal pathogens often mount effective responses to counter the defenses of the host plants. A well-established colonization requires circumventing and overcoming host defense responses such as generation of ROS, basically via hiding, detoxification and inhibition [33]. ROS accumulation is considered as one of the primary responses to *Z. tritici,* starting in the apoplast during the biotrophic stage and spreading to the entire leaf tissue at the late necrotrophic stage. Therefore, *Z. tritici* expresses a number of ROS-scavenging proteins and CWDEs that are known to aid invasion into plant cells by hydrolyzing the plant cell wall polymers. The increased expression of CWDEs may also play a role, although not essential, in nutrient acquisition in *Z. tritici* during the necrotrophic growth [4]. The ability to adapt to the oxidative stress, interfere with host cell structure, and overcome other host defense responses such as expression of apoplastic PR proteins probably results in compatibility with the host wheat. On the other hand, it seems that the resistant plant deploys a highly stressful apoplastic environment to put pressure on the pathogen, as evidenced by the active signaling, transport, and cell wall remodeling in *Z. tritici* during the incompatible interaction. The fungal pathogens are known to sense the stress by rapid signal transduction contributing to the regulation of stress functions including glycerol accumulation and cell wall remodeling [34].

The hypothesis of significance of anti-oxidative stress mechanisms in *Z. tritici* colonization was tested by deletion of a gene encoding YAP1 transcription factor regulating expression of antioxidants. The ability to defend against oxidative stress *in vitro* was partially compromised in *YAP1*-dirupted mutant, which, however, showed normal virulence as the wild type strain, suggesting a non-essential role of *YAP1* in *Z. tritici* pathogenicity and functional redundancy of antioxidants. Similarly, disruption of a *YAP1* homolog did not affect the virulence of the phytopathogenic fungus *Cochliobolus heterostrophus* [35] or *Fusarium graminearum* [36]. These findings highlight the sophisticated mechanisms of the involvement of YAP-mediated ROS detoxification in pathogenicity in fungi kingdom. Further investigations on anti-oxidative stress are highly required to elucidate its clear role in *Z. tritici* pathogenicity.

## Conclusions

We have reported an extensive survey of leaf apoplastic proteome in resistant and susceptible wheat in response to *Z. tritici*. The results collectively demonstrate that plant resistance to *Z. tritici* is correlated with rapid activation of responses at proteome level, including enhanced carbohydrate metabolism, cell wall reinforcement and remodeling, production of PR proteins in the apoplast, and generation of a stressful apoplastic environment. On the other hand, the fungus has to interfere with host cell wall and overcome host defenses and stress at the biotrophic stage, for example, by detoxifying ROS and producing CWDEs, to achieve a successful colonization. Taken together, our work provides the valuable insights into STB resistance and *Z. tritici in planta* proteome, which form a fundamental and prerequisite step for the further research of plant apoplastic immunity and *Z. tritici* pathogenicity.

## Methods

### Plant growth, fungal inoculation and extraction of AWF

Growth of wheat cultivars Sevin (susceptible) and Stakado (resistant), preparation of the inoculum of *Z. tritici* isolate IPO323, and inoculation were preformed as described [8]. Separate control plants were mock inoculated with water. Approximately 60 leaves were collected from four separate pots, serving as one biological replicate. Three biological replications were harvested separately at 5 and 14 dai prior to the extraction of AWF by water as described [37]. The remaining leaf samples were ground into fine powder in liquid nitrogen. The collected AWF and leaf powder were stored at −80°C until use.

### Malate dehydrogenase assay

Total soluble protein was extracted from the leaf powder in 50 mM phosphate buffer, pH 7.5. MDH activity assay was performed to assess the contamination of AWF by intracellular proteins. Five μL of AWF or total leaf protein extract was added to 200 μL of the reaction buffer containing 0.17 mM oxalacetic acid, 0.094 mM β-NADH disodium salt and 0.1 M phosphate buffer, pH 7.5 [14]. The change of absorbance at 340 nm was monitored for 5 min in a spectrophotometer.

### Shotgun proteomics analysis of AWF

Protein concentration in AWF was determined by Bio-Rad Protein Assay (Bio-Rad) with bovine serum albumin as standard. Fifty micrograms protein was precipitated by 5 volumes of 10% TCA (w/v) in acetone at −20°C overnight. The protein pellet was washed three times in cold 100% acetone followed by solubilization in the buffer containing 6 M urea and 2 M thiourea. Protein samples were treated with 10 mM DTT for 30 min at room temperature and 40 mM iodoacetamide for 30 min in the dark prior to digestion with trypsin (2%, w/w) at 37°C overnight. The resulting peptides were purified on Poros Oligo R3 microcolumn and vacuum-dried prior to amino acid analysis using a Biochrom 30+ Amino Acid Analyzer (Biochrom, UK). Six micrograms peptides from each biological replicate of inoculated and control samples were labeled with iTRAQ® 4-plex (Applied Biosystems) according to the manufacturer’s protocol (114 for the control at 5 dai, 115 for the inoculated sample at 5 dai, 116 for the control at 14 dai, 117 for the inoculated sample at 14 dai). Labeled peptides were combined and desalted on Poros Oligo R3 microcolumn.

The labeled peptides were fractionated using hydrophilic interaction liquid chromatography (HILIC) fractionation and analyzed by liquid chromatography (LC)-MS/MS as described [10] with modifications. Briefly, isobaric-labeled peptides obtained from biological replicate 1 were fractionated on a TSKGel Amide 80 HILIC-HPLC column by using the Agilent 1200 microHPLC instrument. Samples were suspended in solvent B (90% acetonitrile and 0.1% trifluoroacetic acid), and peptides were eluted at 6 μL/min by decreasing the solvent B concentration (100 − 60%) over 26 min. Fractions were collected and lyophilized.

Peptides from biological replicate 1 were resuspended in 0.1% formic acid and separated by reversed-phase liquid chromatography on a Reprosil-Pur C18 (3 μC; Dr. Maisch GmbH - Ammerbuch, Germany) column (22 cm x 100 μm inner diameter, in-house packed). The chromatographic gradient was 0 − 34% solvent B (90% acetonitrile and 0.1% formic acid) for 90 min at a flow rate of 300 nL/min. A LTQ-Orbitrap XL mass spectrometer (Thermo Fisher Scientific) was operated in a data-dependent mode automatically switching between MS and MS/MS. A survey MS scan (400–1800 m/z) was acquired in the Orbitrap analyzer with a resolution of 30000 at 400 m/z. The top three most intense ions with a threshold of 5000 were selected for low-resolution CID at normalized collision energy of 35 and high-resolution HCD (normalized collision energy = 55; 7500 resolution at 400 m/z). Peptides from biological replicates 2 and 3 without HILIC fractionation were performed using the same LC configuration with a 120 min gradient but coupled to a LTQ-Orbitrap Velos mass spectrometer. Following a survey MS scan at a resolution of 30000 at 400 m/z, the top seven most intense ions were selected for high-resolution HCD–MS/MS (normalized collision energy = 48; 7500 resolution at 400 m/z). These two biological replicates were run twice using this setup as technical replicates. Raw data were viewed in Xcalibur v2.0.7 (Thermo Fisher Scientific, USA).

Raw MS/MS spectra were processed and quantified using Proteome Discoverer (Version 1.2, Thermo Fisher) software with quantification setup as ratios of 115/114 (inoculated/control at 5 dai) and 117/116 (inoculated/control at 14 dai). Peptide identification was performed with MASCOT (v2.2, Matrix Science Ltd. - London, UK) and Sequest algorithms, searching against a target and decoy TaGI wheat gene index Release 12.0 (released on 18^th^ April, 2010; TC sequences, 93508; ESTs, 128166; ETs, 251; http://compbio.dfci.harvard.edu/tgi/) and DOE Joint Genome Institute gene index for *Z. tritici* (released on 10^th^ September, 2008; 10933 genes; http://genome.jgi-psf.org/Mycgr3) databases. The following parameters were set for searching: 2 missed cleavages, S-carbamidomethyl-cysteine as a fixed modification, oxidation (M), deamidation (N and Q), iTRAQ® reagents (protein N-terminus and Lys side-chain), peptide mass tolerance 10 ppm, and fragment ion mass tolerance 0.5 Da for CID and 0.05 Da for HCD. False discovery rates were obtained using Percolator selecting identifications with a *q*-value ≤ 0.01. Only high-confidence peptide sequences with a Mascot ion score ≥ 23, Sequest Xcorr value > 2.2 and rank 1 were considered for the further analysis. The identified plant and fungal proteins must contain at least two confidentially identified peptides. Protein quantification data were normalized using log2-transformed median. The statistics analysis was performed on the data merged from all biological and technical replicates by using R program. The plant proteins identified in at least two biological replicates and with average ratios ≥ 2 or ≤ 0.5 at either time point were defined as regulated proteins. The fungal proteins identified in at least two biological replicates with quantitative data were shown in Table 1. SignalP (http://www.cbs.dtu.dk/services/SignalP) was performed to examine signal peptides of fungal and wheat regulated proteins. The differential expression profiles of host proteins at two time points were clustered by *k*-means algorithm using Euclidean distance.

### Western blot analysis

Western blotting was performed with three biological replicates as described [38]. Five micrograms proteins from AWF were separated on CriterionTM XT Precast Gels (12% Bis-Tris, Bio-Rad) followed by blotting to nitrocellulose membranes (GE Healthcare). Membranes were blocked followed by incubation with rabbit antibodies against barley PR-1, PR-2 (β-1,3-glucanase), and PR-3 (chitinase). After extensive washes, the membranes were incubated with anti-rabbit secondary antibodies conjugated to horseradish peroxidase (1:2000, Dako, Denmark) and detected using the Immun-Star HRP Substrate Kit (Bio-Rad). Quantification of the signals on the membranes was carried out by using ImageJ program.

### Agrobacterium-mediated targeted gene deletion of *ZtYAP1*

Approx. 700-bp and 1400-bp of flanking fungal genomic DNA were amplified upstream and downstream of *YAP1* (Zt35076) opening reading frame by PCR, respectively, and cloned into vector pCHYG containing the hph cassette conferring resistance to hygromycin [39]. The final plasmid pCHYG-YAP1 was sequenced to confirm the correct insert. The plasmid was transformed to Agrobacterium strain Agl-1 via the freeze-thaw method. Agrobacterium transformation of *Z. tritici* was performed as described [39]. Fungal genomic DNA was isolated and targeted insertion of the T-DNA was initially confirmed by PCR on genomic DNA directed against Hph and *ZtYAP1*. All the primers used for study of fungal mutant are listed in Additional file 5.

### Characterization of *ZtYAP1* mutant

Preparation of spores of *ZtYAP1* mutant and inoculation of 14-day-old wheat cv. Sevin seedling were performed as described above to evaluate fungal pathogenicity. Three independent pots of plant (ten leaves per pot) were prepared for inoculation with the wild type, the mutant or water, serving as three biological replicates. Leaves were photographed at 10, 12 and 15 days. Infected leaves were harvested at 21 dai. The spores were washed out from leaves and counted under microscope.

Sensitivity assay to oxidative stress was conducted by applying a 5 μL droplet of fungal spore suspension onto PDA containing compounds at appropriate concentrations and incubating under constant fluorescent light. Photographs of relative colony densities were taken after 6 days.

## Additional files

Additional file 1: MDH activity in whole leaf extracts and AWF. Additional file 2: List of identified and quantified wheat proteins from leaf AWF of wheat cvs. Stakado and Sevin inoculated with *Z. tritici*
Additional file 3: List of differentially expressed wheat proteins identified from AWF in response to *Z. tritici*. The expression data of the corresponding transcript obtained from the previous RNA-seq dataset are included. Additional file 4: Expression of the genes encoding identified fungal proteins. The expression data are obtained from the previous RNA-seq analysis of *Z. tritici*-infected wheat (cv. Sevin) at 4, 10 and 13 days. Additional file 5: Primers used in the study.

## Abbreviations

AWF: Apoplastic washing fluid
CWDEs: Cell-wall-degrading enzymes
Dai: Days after inoculation
HILIC: Hydrophilic interaction liquid chromatography
iTRAQ: Isobaric tags for relative and absolute quantitation
LC: Liquid chromatography
LSPs: Leaderless secreted proteins
MDH: Malate dehydrogenase
MS: Mass spectrometry
PDA: Potato dextrose agar
PR: Pathogenesis-related
ROS: Reactive oxygen species
STB: Septoria tritici blotch

## References

[1] Keon J, Antoniw J, Carzaniga R, Deller S, Ward JL, Baker JM (2007). Transcriptional adaptation of *Mycosphaerella graminicola* to programmed cell death (PCD) of its susceptible wheat host. Mol Plant Microbe Interact.

[2] Rudd JJ, Kanyuka K, Hassani-Pak K, Derbyshire M, Andongabo A, Devonshire J (2015). Transcriptome and metabolite profiling the infection cycle of *Zymoseptoria tritici* on wheat (*Triticum aestivum*) reveals a biphasic interaction with plant immunity involving differential pathogen chromosomal contributions, and a variation on the hemibiotrophic lifestyle definition. Plant Physiol.

[3] Marshall R, Kombrink A, Motteram J, Loza-Reyes E, Lucas J, Hammond- Kosack KE (2011). Analysis of two in *planta* expressed LysM effector homologs from the fungus *Mycosphaerella graminicola* reveals novel functional properties and varying contributions to virulence on wheat. Plant Physiol.

[4] Yang F, Li WS, Jørgensen HJ (2013). Transcriptional reprogramming of wheat and the hemibiotrophic pathogen *Septoria tritici* during two phases of the compatible interaction. PLoS One.

[5] Pechanova O, Hsu CY, Adams JP, Pechan T, Vandervelde L, Drnevich J (2010). Apoplast proteome reveals that extracellular matrix contributes to multistress response in poplar. BMC Genomics.

[6] Lee WS, Rudd JJ, Hammond-Kosack KE, Kanyuka K (2014). *Mycosphaerella graminicola* LysM effector-mediated stealth pathogenesis subverts recognition through both CERK1 and CEBiP homologues in wheat. Mol Plant Microbe Interact.

[7] Rudd JJ, Keon J, Hammond-Kosack KE (2008). The Wheat mitogen- activated protein kinases TaMPK3 and TaMPK6 are differentially regulated at multiple levels during compatible disease interactions with *Mycosphaerella graminicola*. Plant Physiol.

[8] Shetty NP, Kristensen BK, Newman MA, Møller K, Gregersen PL, Jørgensen HJ (2003). Association of hydrogen peroxide with restriction of *Septoria tritici* in resistant wheat. Physiol Mol Plant Pathol.

[9] Shetty NP, Mehrabi R, Lütken H, Haldrup A, Kema GH, Collinge DB (2007). Role of hydrogen peroxide during the interaction between the hemibiotrophic fungal pathogen *Septoria tritici* and wheat. New Phytol.

[10] Yang F, Melo-Braga MN, Larsen MR, Jørgensen HJ, Palmisano G (2013). Battle through signaling between wheat and the fungal pathogen *Septoria tritici* revealed by proteomics and phosphoproteomics. Mol Cell Proteomics.

[11] Kema GH, van der Lee TA, Mendes O, Verstappen EC, Lankhorst RK, Sandbrink H (2008). Large-scale gene discovery in the septoria tritici blotch fungus *Mycosphaerella graminicola* with a focus on *in planta* expression. Mol Plant Microbe Interact.

[12] Toone WM, Morgan BA, Jones N (2001). Redox control of AP-1- like factors in yeast and beyond. Oncogene.

[13] Lin CH, Yang SL, Chung KR (2009). The YAP1 homolog-mediated oxidative stress tolerance is crucial for pathogenicity of the necrotrophic fungus *Alternaria alternata* in citrus. Mol Plant Microbe Interact.

[14] Husted S, Schjoerring JK (1995). Apoplastic pH and ammonium concentration in leaves of *Brassica napus* L. Plant Physiol.

[15] Mattsson M, Schjørring JK (2003). Senescence-induced changes in apoplastic and bulk tissue ammonia concentrations of ryegrass leaves. New Phytol.

[16] Floerl S, Druebert C, Majcherczyk A, Karlovsky P, Kües U, Polle A (2008). Defence reactions in the apoplastic proteome of oilseed rape (*Brassica napus* var. *napus*) attenuate *Verticillium longisporum* growth but not disease symptoms. BMC Plant Biol.

[17] Floerl S, Majcherczyk A, Possienke M, Feussner K, Tappe H, Gatz C (2012). *Verticillium longisporum* infection affects the leaf apoplastic proteome, metabolome, and cell wall properties in *Arabidopsis thaliana*. PLoS One.

[18] Delaunois B, Colby T, Belloy N, Conreux A, Harzen A, Baillieul F (2013). Large-scale proteomic analysis of the grapevine leaf apoplastic fluid reveals mainly stress-related proteins and cell wall modifying enzymes. BMC Plant Biol.

[19] Witzel K, Shahzad M, Matros A, Mock HP, Muhling KH (2011). Comparative evaluation of extraction methods for apoplastic proteins from maize leaves. Plant Methods.

[20] Djordjevic MA, Oakes M, Li DX, Hwang CH, Hocart CH, Gresshoff PM (2007). The glycine max xylem sap and apoplast proteome. J Proteome Res.

[21] Soares NC, Francisco R, Ricardo CP, Jackson PA (2007). Proteomics of ionically bound and soluble extracellular proteins in *Medicago truncatula* leaves. Proteomics.

[22] Casasoli M, Spadoni S, Lilley KS, Cervone F, De Lorenzo G, Mattei B (2008). Identification by 2-D DIGE of apoplastic proteins regulated by oligogalacturonides in *Arabidopsis thaliana*. Proteomics.

[23] Köllner TG, Schnee C, Li S, Svatoš A, Schneider B, Gershenzon J (2008). Protonation of a neutral (S)-β-bisabolene intermediate is involved in (S)-β-macrocarpene formation by the maize sesquiterpene synthases TPS6 and TPS11. J Biol Chem.

[24] Schmelz EA, Kaplan F, Huffaker A, Dafoe NJ, Vaughan MM, Ni X (2011). Identity, regulation, and activity of inducible diterpenoid phytoalexins in maize. Proc Natl Acad Sci U S A.

[25] Agrawal GK, Jwa NS, Lebrun MH, Job D, Rakwal R (2010). Plant secretome: unlocking secrets of the secreted proteins. Proteomics.

[26] Chivasa S, Simon W, Yu XL, Yalpani N, Slabas A (2005). Pathogen elicitor-induced changes in the maize extracellular matrix proteome. Proteomics.

[27] Cheng FY, Blackburn K, Lin YM, Goshe MB, Williamson JD (2009). Absolute protein quantification by LC/MSE for global analysis of salicylic acid-induced plant protein secretion responses. J Proteome Res.

[28] Yang F, Jensen JD, Svensson B, Jørgensen HJ, Collinge DB, Finnie C (2012). Secretomics identifies *Fusarium graminearum* proteins involved in the interaction with barley and wheat. Mol Plant Pathol.

[29] Bolton MD (2009). Primary metabolism and plant defense-fuel for the fire. Mol Plant Microbe Interact.

[30] Underwood W (2012). The plant cell wall: a dynamic barrier against pathogen invasion. Frontier Plant Sci.

[31] Song Y, Zhang C, Ge W, Zhang Y, Burlingame AL, Guo Y (2011). Identification of NaCl stress-responsive apoplastic proteins in rice shoot stems by 2D-DIGE. J Proteomics.

[32] Le Roch KG, Johnson JR, Florens L, Zhou Y, Santrosyan A, Grainger M (2004). Global analysis of transcript and protein levels across the *Plasmodium falciparum* life cycle. Genome Res.

[33] Doehlemann G, Hemetsberger C (2013). Apoplastic immunity and its suppression by filamentous plant pathogens. New Phytol.

[34] Brown AJ, Budge S, Kaloriti D, Tillmann A, Jacobsen MD, Yin Z (2014). Stress adaptation in a pathogenic fungus. J Exp Biol.

[35] Lev S, Hadar R, Amedeo P, Baker S, Yoder OC, Horwitz BA (2005). Activation of an AP-1-like transcription factor of the maize pathogen *Cochliobolus heterostrophus* in response to oxidative stress and plant signals. Eukaryot Cell.

[36] Montibus M, Ducos C, Bonnin-Verdal M-N, Bormann J, Ponts N, Richard- Forget F (2013). The bZIP transcription factor Fgap1 mediates oxidative stress response and trichothecene biosynthesis but not virulence in *Fusarium graminearum*. PLoS One.

[37] Shetty NP, Jensen JD, Knudsen A, Finnie C, Geshi N, Blennow A (2009). Effects of β-1,3-glucan from *Septoria tritici* on structural defence responses in wheat. J Exp Bot.

[38] Yang F, Jensen JD, Svensson B, Jørgensen HJ, Collinge DB, Finnie C (2010). Analysis of early events in the interaction between *Fusarium graminearum* and the susceptible barley (*Hordeum vulgare)* cultivar Scarlett. Proteomics.

[39] Motteram J, Küfner I, Deller S, Brunner F, Hammond-Kosack KE, Nürnberger T (2009). Molecular characterization and functional analysis of MgNLP, the sole NPP1 domain-containing protein, from the fungal wheat leaf pathogen *Mycosphaerella graminicola*. Mol Plant Microbe Interact.

