# Supplementary material for: Unraveling incompatibility between wheat and the fungal pathogen Zymoseptoria tritici through apoplastic proteomics
Source: BMC Genomics. 2015 May 8;16(1):362. doi: 10.1186/s12864-015-1549-6 (PMC4423625; doi:10.1186/s12864-015-1549-6)
Supplement: Additional file 1: — MDH activity in whole leaf extracts and AWF. [file 12864_2015_1549_MOESM1_ESM.docx]

**Table S1** **Malate dehydrogenase activity assay**

| Treatments | Stakado | | |  | Sevin | | |
| --- | --- | --- | --- | --- | --- | --- | --- |
|  | MDH activity ratio (apoplast/total,%) | Total protein (μg/mg FW) | Apoplast protein (μg/mg FW) |  | MDH activity ratio (apoplast/total,%) | Total protein (μg/mg FW) | Apoplast protein (μg/mg FW) |
| C 5 d | 1.05 ±0.02 | 2.99±0.33 | 0.058±0.002 |  | 0.61± 0.22 | 3.31±0.16 | 0.06±0.008 |
| I 5 d | 0.69± 0.02 | 2.21±0.47 | 0.06±0.007 |  | 0.59± 0.16 | 2.89±1.01 | 0.067±0.008 |
| C 14 d | 0.53± 0.22 | 2.35±0.4 | 0.076±0.028 |  | 0.47± 0.04 | 1.81±0.1 | 0.072±0.023 |
| I 14 d | 0.29 ±0.07 | 2.22±0.08 | 0.118±0.008 |  | 1.09± 0.51 | 1.53±0.78 | 0.079±0.005 |

Data are the mean of the values of three biological replicates ± SD. The absolute MDH activity was calculated by

the decrease in absorbance at 340 nm/min/(mg protein/g FW). Abbreviations: FW, fresh weight of the wheat

leaves; C, control; I, inoculated sample.
